# Recruitment Strategies in the Integration of Mobile Health Into Sickle Cell Disease Care to Increase Hydroxyurea Utilization Study (meSH): Multicenter Survey Study

**DOI:** 10.2196/48767

**Published:** 2024-04-16

**Authors:** Chinonyelum Nwosu, Hamda Khan, Rita Masese, Judith M Nocek, Siera Gollan, Taniya Varughese, Sarah Bourne, Cindy Clesca, Sara R Jacobs, Ana Baumann, Lisa M Klesges, Nirmish Shah, Jane S Hankins, Matthew P Smeltzer

**Affiliations:** 1 Department of Hematology St. Jude Children's Research Hospital Memphis, TN United States; 2 Duke University Durham, NC United States; 3 University of Illinois at Chicago Chicago, IL United States; 4 Augusta University Augusta, GA United States; 5 Washington University School of Medicine St Louis, MO United States; 6 Medical University of South Carolina Charleston, SC United States; 7 Icahn School of Medicine at Mount Sinai New York, NY United States; 8 RTI International Research Triangle Park, NC United States; 9 Department of Global Pediatric Medicine St. Jude Children's Research Hospital Memphis, TN United States; 10 The University of Memphis Memphis, TN United States

**Keywords:** sickle cell, recruitment, eHealth, multicenter, utilization, strategy, hydroxyurea, mobile health, mhealth, intervention

## Abstract

**Background:**

Hydroxyurea is an evidence-based disease-modifying therapy for sickle cell disease (SCD) but is underutilized. The Integration of Mobile Health into Sickle Cell Disease Care to Increase Hydroxyurea Utilization (meSH) multicenter study leveraged mHealth to deliver targeted interventions to patients and providers. SCD studies often underenroll; and recruitment strategies in the SCD population are not widely studied. Unanticipated events can negatively impact enrollment, making it important to study strategies that ensure adequate study accrual.

**Objective:**

The goal of this study was to evaluate enrollment barriers and the impact of modified recruitment strategies among patients and providers in the meSH study in response to a global emergency.

**Methods:**

Recruitment was anticipated to last 2 months for providers and 6 months for patients. The recruitment strategies used with patients and providers, new recruitment strategies, and recruitment rates were captured and compared. To document recruitment adaptations and their reasons, study staff responsible for recruitment completed an open-ended 9-item questionnaire eliciting challenges to recruitment and strategies used. Themes were extrapolated using thematic content analysis.

**Results:**

Total enrollment across the 7 sites included 89 providers and 293 patients. The study acceptance rate was 85.5% (382/447) for both patients and providers. The reasons patients declined participation were most frequently a lack of time and interest in research, while providers mostly declined because of self-perceived high levels of SCD expertise, believing they did not need the intervention. Initially, recruitment involved an in-person invitation to participate during clinic visits (patients), staff meetings (providers), or within the office (providers). We identified several important recruitment challenges, including (1) lack of interest in research, (2) lack of human resources, (3) unavailable physical space for recruitment activities, and (4) lack of documentation to verify eligibility. Adaptive strategies were crucial to alleviate enrollment disruptions due to the COVID-19 pandemic. These included remote approaching and consenting (eg, telehealth, email, and telephone) for patients and providers. Additionally, for patients, recruitment was enriched by simplification of enrollment procedures (eg, directly approaching patients without a referral from the provider) and a multitouch method (ie, warm introductions with flyers, texts, and patient portal messages). We found that patient recruitment rates were similar between in-person and adapted (virtual with multitouch) approaches (167/200, 83.5% and 126/143, 88.1%, respectively; *P*=.23). However, for providers, recruitment was significantly higher for in-person vs remote recruitment (48/50, 96% and 41/54, 76%, respectively, *P*<.001).

**Conclusions:**

We found that timely adaptation in recruitment strategies secured high recruitment rates using an assortment of enriched remote recruitment strategies. Flexibility in approach and reducing the burden of enrollment procedures for participants aided enrollment. It is important to continue identifying effective recruitment strategies in studies involving patients with SCD and their providers and the impact and navigation of recruitment challenges.

**Trial Registration:**

ClinicalTrials.Gov NCT03380351; https://clinicaltrials.gov/study/NCT03380351

**International Registered Report Identifier (IRRID):**

RR2-10.2196/16319

## Introduction

Sickle cell disease (SCD) is an inherited blood disorder that affects approximately 100,000 individuals in the United States [1]. SCD causes debilitating health issues, including progressive organ damage, acute unplanned severe vaso-occlusive crises (VOCs), and premature death [2]. Treatment with hydroxyurea increases fetal hemoglobin and has other salutary benefits (eg, reduced inflammation) that together function as protection against organ damage and VOCs and increase the rate of patient survival [3]. Despite the proven efficacy and effectiveness of hydroxyurea as a prophylactic treatment in SCD, it is vastly underutilized, a result of a combination of poor prescribing practices among providers and low adherence behavior among patients [4].

In 2016, the National Heart, Lung, and Blood Institute established the Sickle Cell Disease Implementation Consortium (SCDIC), which was tasked with conducting implementation research and interventional studies tailored toward improving the quality of SCD care [5]. One of the interventional studies, the Integration of Mobile Health Into Sickle Cell Disease Care to Increase Hydroxyurea Utilization (meSH) study, addressed the barriers to hydroxyurea uptake at both the provider and patient levels by targeting the multilevel determinants of poor hydroxyurea utilization [6,7]. In meSH, the targeted interventions were delivered using 2 mobile health (mHealth) apps: the patient app, InCharge Health [8], and the provider app, HU Toolbox [8]. In March 2020, only 4 months after the study began recruiting providers and 2 months after the initiation of patient recruitment, the global COVID-19 pandemic emerged in the United States. In response to the pandemic, institutions were forced to modify clinical and research procedures by temporarily suspending all nonessential in-person contact [9]. This rapid procedural change directly impacted all in-person research activities in meSH, which was initially designed to conduct all recruitment in person. The meSH study team quickly adapted recruitment strategies for the ongoing study, eventually meeting enrollment goals.

In general, SCD studies suffer from underrecruitment [10]. SCD disproportionately affects African-Americans, making it imperative that recruitment is intentional. When targeting a historically underrepresented group, there are some recruitment strategies that have been shown to be highly effective, such as having a champion of the study on site to promote recruitment, employing culturally matched staff, using flyers with images that represent and resonate with the group, and providing culturally relevant incentives [11]. These intentional adjustments to the recruitment process help to address some of the common barriers to research for historically underrepresented groups. Recruitment targets must be met to ensure sufficient power to answer research questions, and recruitment strategies can help ensure this goal. However, recruitment strategies in the SCD population are not widely studied [2]. The goal of this study was to evaluate enrollment barriers faced by the meSH study and the impact of modified recruitment strategies for patients and providers in response to a global emergency.

## Methods

### Recruitment Strategies for the meSH Study

The meSH study used the InCharge Health mobile app in an implementation study with three aims: (1) improve patient adherence to hydroxyurea, (2) improve provider hydroxyurea prescribing behaviors, and (3) evaluate barriers and facilitators to implementation of mobile health interventions [6,7]. The meSH study design used a staggered site initiation across 7 sites, with the first 2 sites beginning the enrollment process in September 2019 for providers and in November 2019 for patients. The SCDIC consortium includes clinical sites in academic and nonacademic settings and in both rural and urban settings. Development of this cohort used implementation science principles previously described in detail (ClinicalTrials.Gov NCT03380351) [12].

For our evaluation of meSH recruitment strategies, the eligibility criterion was functioning as the lead study coordinator for a site (all lead study coordinators agreed to participate). For the meSH study itself, the eligibility criteria for providers included being a physician (including a physician in training) or advanced practice provider (ie, nurse practitioner or physician assistant) who cared for at least 1 patient with SCD for an anticipated minimum of 12 months from study enrollment and had access to a smartphone (either Android or iOS) or a computer with internet connectivity (the HU Toolbox app can be accessed via the internet on any device) [6,7]. Eligibility criteria for patients included being aged 15 to 45 years; receiving treatment at or being affiliated with one of the SCDIC sites; speaking English; having a confirmed SCD diagnosis by a hemoglobin fractionation test; owning a smartphone (either Android or iOS); and being treated with hydroxyurea therapy, defined as already receiving hydroxyurea therapy (at least 1 previous prescription for hydroxyurea in the past 3 months) or initiating hydroxyurea therapy (the first prescription must have been written at study enrollment) [6,7].

Enrollment of patients was initially planned to occur in person during routine clinic visits. Enrollment of providers was planned in person, to occur during staff meetings, at clinics, or in the providers’ offices. The method of enrollment, modification of the enrollment method, and refusal reasons were tracked prospectively. Ineligibility was documented during the screening process and captured via CONSORT forms, and reasons for declining were collected by site study coordinators. REDCap (Research Electronic Data Capture; Vanderbilt University), a secure electronic database, was used to administer and store the surveys, survey data, and consent forms.

To better understand the barriers and adaptations to study recruitment, we surveyed the primary research coordinator responsible for recruitment at each of the 7 participating sites with 9 open-ended questions regarding recruitment strategies, adaptations to enrollment methods, and challenges for enrolling patients and providers (Multimedia Appendix 1). The interview guides included were open-ended questions developed to elicit (1) methods used for recruitment, specifically the details of modifications to the original recruitment process listed in the protocol, such as adaptations or deviations from the in-person recruitment process originally planned; (2) temporal variations of each recruitment modality (ie, how long each recruitment strategy was used and when it was changed), (3) reasons for modification of recruitment strategies, and (4) the perception of success for each recruitment strategy. Responses were deidentified and tabulated, and a thematic content analysis was conducted. Responses were first organized inductively by the main domains they addressed and then categorized based on the identified thematic group, allowing for the organic emergence of themes.

We summarized provider and patient participant characteristics (as frequencies and percentages) overall and by site to illustrate site-level differences. Statistical comparisons were made using chi-square tests with an α level of .05. All analyses were conducted using SAS (version 9.4; SAS Institute).

### Ethical Considerations

For deidentified participant data access, the original data for this study will be made available at BioLINCC [13]. The meSH study attained institutional review board (IRB) approval at all 7 participating sites (St. Jude Children’s Research Hospital, 19-0159; Augusta University IRB, 1528751; UIC IRB, 2020-0087; Duke University IRB, Pro00073506; Mount Sinai IRB, STUDY-20-00303; Medical University of South Carolina [MUSC] IRB, Pro00097832; Washington University at St. Louis IRB, 202003150). All patient participants (or their legal guardians) signed informed consent, and all provider participants gave verbal assent before any study procedure.

## Results

### Provider Recruitment and Adapted Recruitment Strategies

All eligible providers in all 7 sites were approached to participate. Of the 104 approached, 89 enrolled, an 86% study acceptance rate. Of the 89 providers enrolled, most were female (65/89, 73%), aged 25 to 44 years (53/89, 60%), White (47/89, 53%), and not Hispanic or Latino (84/89, 94%); most considered themselves SCD specialists [12] (54/89, 61%) (Table 1). The provider recruitment and enrollment duration ranged from 2 to 7 months across all 7 sites. Initially, all sites planned to use an in-person recruitment approach. Some opted to introduce the study via a presentation at regularly scheduled staff meetings, followed by in-person individual provider enrollment. With the onset of the COVID-19 pandemic in March 2020, most sites suspended in-person research activities, which posed recruitment challenges [3]. At that time, recruitment to meSH pivoted from an in-person to a virtual approach. Virtual enrollment of providers was conducted by email. An introductory email was sent by the site study coordinators that detailed the meSH study and inquired about interest in participation. This email was followed by reminder emails every 1 to 2 weeks until recruitment ended in March of 2021. In total, 41 of 54 (76%) providers approached virtually accepted, compared to 48 of 50 (96%) providers who were approached in-person and enrolled (*P*<.001).

The most common challenges site coordinators faced in recruiting providers were a lack of response, a lack of interest due to an impersonal experience or perceived lack of need for intervention, a lack of time in provider schedules, and technical issues with the provider’s phone, such as outdated software that inhibited them from downloading the intervention app.

**Table 1:** Demographics of providers enrolled in the Integration of Mobile Health Into Sickle Cell Disease Care to Increase Hydroxyurea Utilization (meSH) study. The meSH study was a nonrandomized, closed cohort hybrid-effectiveness trial that used staggered site initiation for recruitment. The study was conducted in the sickle cell disease population at 7 academic institutions across the United States from September 2019 to August 2022.

		Site 1 (n=15), n (%)	Site 2 (n=27), n (%)	Site 3 (n=3), n (%)	Site 4 (n=8), n (%)	Site 5 (n=12), n (%)	Site 6 (n=17), n (%)	Site 7 (n=7), n (%)	Total (n=89), n (%)
**Gender**									
	Male	2 (13)	10 (37)	0 (0)	0 (0)	1 (8)	5 (29)	2 (33)	20 (23)
	Female	13 (87)	17 (63)	3 (100)	8 (100)	11 (92)	10 (59)	3 (50)	65 (73)
**Age (years)**									
	25-44	8 (53)	20 (74)	3 (100)	2 (25)	7 (58)	9 (53)	4 (67)	53 (60)
	45-64	6 (40)	7 (26)	0 (0)	4 (50)	3 (25)	6 (35)	2 (33)	28 (32)
	65 and older	0 (0)	0 (0)	0 (0)	2 (25)	2 (17)	1 (6)	0 (0)	5 (6)
**Race**									
	American Indian or Alaska Native	0 (0)	0 (0)	1 (33)	0 (0)	0 (0)	0 (0)	1 (17)	2 (2)
	Asian	2 (13)	7 (26)	0 (0)	0 (0)	2 (17)	5 (29)	0 (0)	16 (18)
	Black or African American	5 (33)	3 (11)	2 (67)	2 (25)	5 (42)	5 (29)	2 (33)	24 (27)
	Native Hawaiian or Pacific Islander	0 (0)	0 (0)	0 (0)	0 (0)	0 (0)	0 (0)	0 (0)	0 (0)
	White	7 (47)	17 (63)	1 (33)	6 (75)	6 (50)	6 (35)	4 (67)	47 (53)
**Ethnicity**									
	Hispanic or Latino	1 (7)	0 (0)	0 (0)	0 (0)	0 (0)	1 (6)	1 (17)	3 (3)
	Not Hispanic or Latino	14 (93)	27 (100)	3 (100)	8 (100)	12 (100)	15 (88)	5 (83)	84 (94)
**Level of SCD expertise**									
	Level I: unengaged	0 (0)	0 (0)	0 (0)	0 (0)	0 (0)	0 (0)	0 (0)	0 (0)
	Level II: willing	4 (27)	3 (11)	0 (0)	2 (25)	1 (8)	7 (41)	1 (17)	18 (21)
	Level III: willing high-volume	1 (7)	7 (26)	0 (0)	0 (0)	1 (8)	4 (24)	1 (17)	14 (16)
	Level IV: expert	9 (60)	17 (63)	3 (100)	6 (75)	10 (83)	5 (29)	4 (67)	54 (61)

### Patient Recruitment

**Figure 1:**
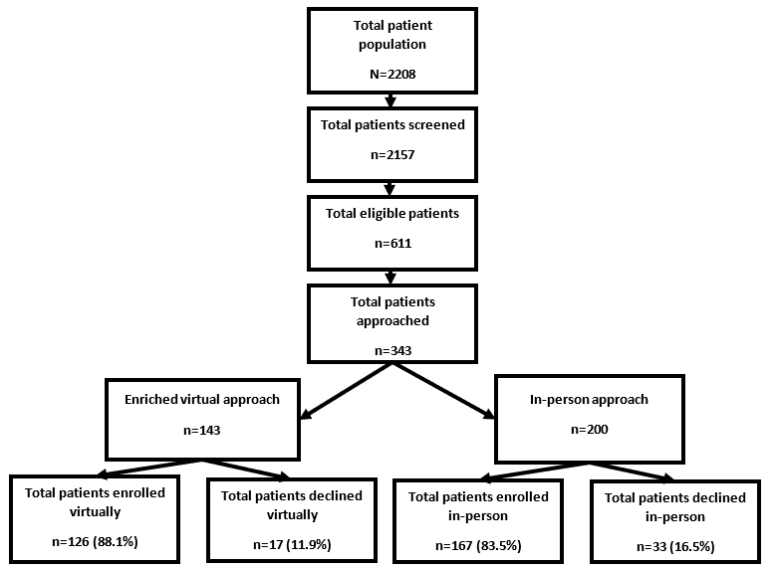
A CONSORT (Consolidated Standards of Reporting Trials) diagram of patient recruitment for the Integration of Mobile Health Into Sickle Cell Disease Care to Increase Hydroxyurea Utilization (meSH) study, including the total numbers of patients approached and enrolled virtually and in person. The meSH study was a nonrandomized, closed cohort hybrid-effectiveness trial that used staggered site initiation for recruitment. The study was conducted in the sickle cell disease population at 7 academic institutions across the United States from September 2019 to August 2022.

The total patient population treated with hydroxyurea across all 7 sites was 2208 patients. Of those, 2157 (97.6%) patients were screened for eligibility. Of the 2157 individuals screened, 611 (28.3%) patients were eligible for the study, and 343 (56%) were approached for enrollment (Figure 1).

Of the 343 eligible patients approached, 293 accepted participation and were successfully enrolled across all 7 sites, an 85.4% participation rate. Among those enrolled, most patients were aged between 26 to 45 years (164/293, 56%), were Black or African American (283/293, 97%), were not Hispanic or Latino (279/293, 95%), were never married (242/293, 83%), had a high school degree or some college or vocational training (176/293, 60%), were not employed (115/293, 39%), and had a household income of $25,000 or less (166/293, 57%) (Table 2). In total, 126 of 143 (88%) patients virtually approached were enrolled, compared to 167 of 200 (83.5%) patients who were approached in person (*P*=.23) (Figure 1).

Although slightly lower than the projected 46 patients per site (322 total patients), our total patient participant sample size of 293 met the requirements of the a priori statistical design [5].

A total of 1546 patients were screened and deemed ineligible across all 7 sites, with the most common reason for ineligibility being “not treated with hydroxyurea” for 49.8% of patients. A total of 50 patients who were approached declined participation. Reasons for declining participation included not being interested in research, not having time to participate in research, and not being interested in the app because of already having a reminder system or thinking the app would not help them.

**Table 2:** Demographics of patients enrolled in the Integration of Mobile Health Into Sickle Cell Disease Care to Increase Hydroxyurea Utilization (meSH) study.

		Site 1 (n=48), n (%)	Site 2 (n=46), n (%)	Site 3 (n=47), n (%)	Site 4 (n=41), n (%)	Site 5 (n=45), n (%)	Site 6 (n=47), n (%)	Site 7 (n=19), n (%)	Total (n=293), n (%)
**Age (years)**									
	15-17	0 (0)	10 (22)	0 (0)	5 (12)	8 (18)	0 (0)	0 (0)	23 (8)
	18-25	22 (46)	28 (61)	12 (26)	18 (44)	20 (44)	4 (9)	2 (10)	106 (36)
	26-45	26 (54)	8 (17)	35 (74)	18 (44)	17 (36)	43 (92)	17 (90)	164 (56)
**Gender**									
	Male	18 (38)	24 (52)	27 (57)	15 (37)	29 (64)	18 (38)	10 (53)	141 (48)
	Female	30 (63)	22 (48)	20 (43)	26 (63)	16 (36)	29 (62)	9 (47)	151 (52)
**Race^a^**									
	Black or African American	48 (100)	46 (100)	47 (100)	41 (100)	41 (91)	47 (100)	14 (74)	283 (97)
	Other	0 (0)	0 (0)	1 (2)	0 (0)	4 (9)	0 (0)	3 (16)	8 (3)
	Missing	0 (0)	0 (0)	0 (0)	0 (0)	0 (0)	0 (0)	2 (11)	2 (1)
**Ethnicity**									
	Hispanic or Latino	0 (0)	0 (0)	1 (2)	0 (0)	1 (2)	0 (0)	9 (47)	11 (4)
	Not Hispanic or Latino	48 (100)	46 (100)	46 (98)	41 (100)	44 (98)	45 (96)	10 (53)	279 (95)
	Missing	0 (0)	0 (0)	0 (0)	0 (0)	0 (0)	2 (4)	0 (0)	2 (1)
**Marital** **status**									
	Married or living as married	6 (13)	1 (2)	10 (21)	0 (0)	4 (9)	5 (11)	4 (21)	30 (10)
	Never married	39 (81)	41 (89)	36 (77)	33 (83)	41 (91)	37 (79)	15 (79)	242 (83)
	Divorced, separated, or widowed	2 (4)	3 (7)	1 (2)	2 (5)	0 (0)	3 (6)	0 (0)	11 (4)
	Missing	1 (2)	1 (2)	0 (0)	5 (13)	0 (0)	2 (4)	0 (0)	10 (3)
**Education**									
	Less than high school or some high school	4 (8)	15 (33)	3 (6)	6 (15)	9 (20)	4 (9)	2 (11)	43 (15)
	High school graduate or some college or vocational training	28 (58)	24 (52)	33 (70)	21 (51)	26 (58)	31 (66)	13 (68)	176 (60)
	College graduate or greater	16 (33)	6 (13)	11 (23)	9 (22)	10 (22)	10 (21)	4 (21)	66 (23)
	Missing	0 (0)	1 (2)	0 (0)	5 (12)	0 (0)	2 (4)	0 (0)	8 (3)
**Employment**									
	Employed	18 (38)	13 (28)	18 (38)	10 (24)	16 (36)	13 (28)	7 (37)	95 (32)
	Not employed by choice (homemaker, student, retired)	16 (33)	23 (50)	3 (6)	8 (20)	14 (31)	7 (15)	4 (21)	75 (26)
	Not employed for other reasons (laid off, sick leave, maternity leave, unemployed, disabled)	14 (29)	9 (20)	26 (55)	18 (44)	15 (33)	25 (53)	8 (42)	115 (39)
	Missing	0 (0)	1 (2)	0 (0)	5 (12)	0 (0)	2 (4)	0 (0)	8 (3)
**Household** **income**									
	$25,000 or less	24 (50)	19 (41)	35 (74)	26 (63)	22 (49)	32 (68)	8 (42)	166 (57)
	$25,001-$50,000	6 (13)	18 (39)	9 (19)	7 (17)	8 (18)	9 (19)	3 (16)	60 (20)
	≥$50,001	12 (25)	5 (11)	2 (4)	3 (7)	10 (22)	3 (6)	5 (26)	40 (14)
	Missing	6 (13)	4 (9)	1 (2)	5 (12)	5 (11)	3 (6)	3 (16)	27 (9)

^a^Individuals could select more than one race.

### Patient Recruitment Strategy Adaptations

Patient recruitment challenges fell into 3 themes: lack of interest in participating in research, lack of resources (staff and space), and lack of proper documentation to verify study eligibility. Specifically, challenges included difficulty verifying hydroxyurea status, technical issues such as nonworking cell phones or phone storage issues, inability to reach patients due to reduced clinic flow as patients opted for telehealth or telemedicine visits, wrong or disconnected phone numbers, and lack of interest in research participation.

Patient recruitment was initially designed to be conducted fully in person. In-person recruitment involved screening and approaching eligible patients during a regularly scheduled, nonacute clinic visit. Due to the COVID-19 pandemic restrictions for in-person research activities, virtual enrollment was initiated. The sites most affected by clinic shutdowns were the first 2 sites, as previously reported [9]. Although clinic lockdowns were no longer in effect when the remaining 5 sites opened enrollment, these sites were also affected by long-term changes related to the pandemic (eg, staff resignation, remote working). The subsequent sites, however, benefited from institutional adjustments to the ongoing pandemic and amended their protocols to include remote recruitment before initiating enrollment. The approach for virtual enrollment included telephone and Zoom (Zoom Video Communications, Inc) calls. Research coordinators at some sites attempted to join clinicians during telehealth patient visits. However, leveraging telehealth clinical visits to conduct study enrollment was unsuccessful. It was not feasible for some sites to add extra time to telehealth clinical visits for research purposes because of already strained logistic issues with establishing connections and providers feeling overwhelmed with managing a new system. One site used the electronic health record (EHR) to send patients secure messages through the patient portal. This approach was intended to create study awareness among eligible participants and to prime them to expect a recruitment phone call. Other sites that transitioned to virtual enrollment contacted patients via phone calls and text messages.

While most of the challenges coordinators faced were during the enrollment phase, coordinators also expressed challenges during the screening portion of the recruitment phase. One challenge was verifying eligibility due to poor documentation of hydroxyurea use in the patients’ charts. In some cases, coordinators found it difficult to search the EHR to find a definitive answer to whether participants were taking hydroxyurea and, if they were, the start and stop dates. Multiple study coordinators reported having a difficult time finding patients that were interested in participating in the study. When asked, most participants gave vague answers such as “just being uninterested in research.” At the same time, some gave more specific answers such as “not having time for study-related activities” or “participating in too many studies (research fatigue).” To overcome these challenges, some sites modified their screening process to optimize identifying eligible patients. For instance, one site sought the recommendations of the hematologists for participants to enroll and for permission to approach them. However, this strategy of seeking provider input ultimately was not sustainable as it consumed too much time for the research coordinators and providers. To save time, the research coordinators sent courtesy messages to the SCD providers to let them know which patients they planned to approach for enrollment. Providers accepted this approach as they believed it led to the least interference with clinic flow and impact on their time.

During the enrollment phase, some coordinators also mentioned a lack of physical and human resources, such as a shortage of research staff (as some resigned during the COVID-19 pandemic) and a lack of space to conduct research, as areas of their clinics and buildings reduced space for research activities due to restrictions on in-person contact. Multiple coordinators mentioned that the biggest hindrance was a need for more staff to help support study implementation. The reasons for staff shortage varied. Still, one common theme was the presence of other studies competing for the time and focus of a limited number of study coordinators. Coordinators adapted their recruitment strategies to overcome challenges. Coordinators with working space restrictions began to rely heavily on remote methods (eg, phone, text message, telehealth) for recruitment instead of in-person methods and successfully sustained recruitment activities. Those with technology issues worked closely with their IT departments to reach feasible solutions, including a tip sheet provided by the IT department to the coordinators for reference for future problems. For sites experiencing staffing shortages and competing studies, coordinators prioritized the enrollment goals of the meSH study while more staff were being hired.

## Discussion

In a multicenter study that tested multilevel mHealth interventions to improve hydroxyurea utilization, recruitment strategies had to be modified and adapted to conform to unforeseen circumstances, which included a global pandemic and its downstream effects, including staff shortages and research space limitations. Despite these unexpected barriers, research staff were nimble in adapting and introducing new recruitment strategies, ultimately leading to sufficient study accrual. We found that an in-person recruitment strategy was more successful than a virtual approach for recruiting providers. For recruiting eligible patients, both in-person and virtual strategies worked well.

The study team launched provider enrollment with a study presentation, allowing providers to ask questions and generate interest in the study. Using a regularly scheduled staff meeting to facilitate provider recruitment helped to eliminate the issue of addressing providers’ busy schedules. Virtual enrollment for patients was effective, with multiple touch points being the best perceived method by study coordinators. Sites that were able to reach out to participants in multiple ways to promote awareness of the study and prime them for future recruitment contact reported fewer obstacles in enrolling patients according to study coordinators. Switching to remote enrollment and using multitouch contact with patients allowed for successful recruitment.

Our study demonstrates that both patients and providers can be successfully enrolled to research using remote methods. However, remote enrollment of patient participants in this study was often paired with additional strategies that offer the familiar human touch. This is one factor that might have aided our successful remote recruitment, since many potential participants were previously engaged and warmly introduced to the study (eg, by receiving a message from the patient portal or being told about the study by a flyer or clinic staff), as opposed to an impersonal contact (ie, an unplanned phone call received from an unfamiliar person). Sites attempted warm contacts in a variety of ways, including text messages, clinic staff, and flyers posted around the clinic.

Compared with other studies that have looked at effective recruitment strategies for enrolling individuals with SCD, our study supports the use of both virtual and in-person research recruitment. Using multiple recruitment strategies could be the most desirable approach to successfully recruit patients [14], such as the multitouch contact approach. With the rise of the pandemic and other factors that may make in-person enrolling difficult, a focus on whether virtual enrollment can be successful has emerged. Our study supports the findings of others, who have found that virtual enrollment can be a successful strategy for recruiting patients with other diseases [15].

A limitation of this paper is that the method used to gather the coordinators’ opinions was not an exhaustive exploration of barriers. In addition, we did not assess facilitators to study participation, which is another important aspect of optimizing participation. Future studies should expound on and further enhance collection of these data using a mixed methods approach to characterize facilitators and barriers, as well as strategies to overcome them, systematically.

Another limitation of this paper is that the coordinators were asked about their perceptions of the benefits and efficiency of the recruitment strategies after the recruitment and enrollment phases were completed; therefore, the responses could be subject to recall bias. The small enrollment goal at each site is also a potential limitation. Enrollment was successful across the sites, but the enrollment goals were relatively modest, making it impossible to conclude that these challenges would have been overcome in larger studies. Additionally, due to the time period, it is not possible to separate our findings from the influence of the burden of responding to COVID-19.

In conclusion, fast adaptation of recruitment strategies was possible in response to the COVID-19 pandemic with remote enrollment combined with multitouch recruitment strategies leading to successful study accrual of patients with SCD. Engaging providers in research can be challenging. Future researchers should continue to identify and test effective recruitment strategies, both in person and remote, for provider participants. Additionally, it will be important to continue identifying effective recruitment strategies in studies involving patients with SCD and their providers and the impact and navigation of recruitment challenges.

## Appendix

Multimedia Appendix 1 Coordinator recruitment experience questions.

Multimedia Appendix 2 Sickle Cell Disease Implementation Consortium members.

## Abbreviations

CONSORT: Consolidated Standard of Reporting Trials
EHR: electronic health record
meSH: Integration of Mobile Health Into Sickle Cell Disease Care to Increase Hydroxyurea Utilization
mHealth: mobile health
REDCap: Research Electronic Data Capture
SCD: sickle cell disease
SCDIC: Sickle Cell Disease Implementation Consortium
VOC: vaso-occlusive crisis
