# Supplementary material for: Recruitment Strategies in the Integration of Mobile Health Into Sickle Cell Disease Care to Increase Hydroxyurea Utilization Study (meSH): Multicenter Survey Study
Source: JMIR Form Res. 2024 Apr 16;8:e48767. doi: 10.2196/48767 (PMC11061784; doi:10.2196/48767)
Supplement: Multimedia Appendix 1 [file formative_v8i1e48767_app1.docx]

Multimedia Appendix 1

Coordinator Recruitment Experience Questions

1. What was your recruitment plan? How exactly did you recruit? Which tactics and activities did you employ?
2. How long did your recruitment phase last?
3. Were there any recruitment challenges? If so, what were they?
4. Did anything change related to how you recruited for the study? If so, what and why?
5. How did you manage or overcome the challenges of recruitment, if any?
6. Were there any adaptations made to the study? For instance, did you have to change how you recruited due to the pandemic? What exactly changed?
7. Any other reason, besides the pandemic, that made you adapt or change how you recruited for the study?
8. If you made any changes, were they successful? In what way? Please explain.
9. What were the reasons eligible participants declined (not including “unwilling to have phone app downloaded” nor “unwilling to attend all study visits”)? Please list them all
