# Supplementary material for: Recruitment Strategies in the Integration of Mobile Health Into Sickle Cell Disease Care to Increase Hydroxyurea Utilization Study (meSH): Multicenter Survey Study
Source: JMIR Form Res. 2024 Apr 16;8:e48767. doi: 10.2196/48767 (PMC11061784; doi:10.2196/48767)
Supplement: Multimedia Appendix 2 [file formative_v8i1e48767_app2.docx]

Multimedia Appendix 2

Sickle Cell Disease Implementation Consortium Members

ST JUDE CHILDREN’S RESEARCH HOSPITAL

Jane S. Hankins, MD, MS

Jason Hodges, PhD, MA

Yvonne Carroll, RN, JD

Lisa Klesges, PhD, MS

Hamda Khan, MA

Matthew Smeltzer, PhD, MS

Chinonyelum Nwosu, MPH

James Gurney, PhD

Jerlym Porter, PhD, MPH

Nicole Alberts, PhD

Reginald French

Sherif Badawy, MD, MS, MBBCh

Michael DeBaun, MD, MPH

Guolian Kang, PhD

Jeremie Estepp, MD

Winfred Wang, MD

Curtis Owens, MD

Margaret Debon, PhD

Ray Osarogiagbon, MD

Marquita Nelson, MD

UNIVERSITY OF CALIFORNIA IN SAN FRANCISCO

Marsha Treadwell, PhD

Elliott Vichinsky, MD

Ted Wun, MD

Michael Potter, MD

Danielle Hessler, PhD

Ward Hagar, MD

Anne Marsh, MD

Lynne Neumayr, MD

MEDICAL UNIVERSITY OF SOUTH CAROLINA

Cathy Melvin, PhD

Julie Kanter, MD

Shannon Phillips, PhD, RN

Robert Adams, MD

Martina Mueller, PhD

DUKE UNIVERSITY

Nirmish Shah, MD

Paula Tanabe, PhD, MSN

Hayden Bosworth, PhD

George Jackson, PhD

Fred Johnson, MBA

Rachel Richesson, PhD

Janet Prvu-Bettger, ScD

WASHINGTON UNIVERSITY

Allison King, MD, PhD

Ana Baumann, PhD

Cecilia Calhoun, PhD

AUGUSTA UNIVERSITY

Abdullah Kutlar, MD

Robert Gibson, PhD

Angie Snyder, PhD

Maria Fernandez, PhD

Richard Lottenberg, MD

MT SINAI

Lynne D. Richardson, MD

Jeffrey Glassberg M.D. M.A.

Jena Simon, MS, APRN-BC

Nicholas G. Genes, MD, PhD

George T. Loo, DrPH

Jason S. Shapiro, MD, MA

Kimberly Souffront PhD, FNP-BC, RN

Cindy Clesca, MA

Elizabeth Linton, MPH

Gery Ryan PhD, MA

RTI INTERNATIONAL

Barbara L Kroner, PhD

Lucia Rojas-Smith, DrPH

Tabitha Hendershot, BA

Lisa DiMartino, PhD, MPH

Sara Jacobs, PhD

Whitney Battestilli, BA

Donald Brambilla, PhD

NHLBI

Sharon M Smith, PhD

William P. Tonkins, Dr. PH, J.D.

Marlene Peters-Lawrence, BSN, RN

Cheryl Boyce, PhD

Whitney Barfield, PhD

LURIE CHILDRENS

Alexis Thompson, MD

UNIVERSITY OF ILLINOIS AT CHICAGO

**Principal Investigators**

Victor Gordeuk, MD -- a

Melissa Gutierrez, MS – i, j

Jana Hirschtick, PhD – i, j

Lewis Hsu, MD, PhD -- b

Jerry Krishnan, MD, PhD -- c

Nadew Sebro, MD -- j

Larissa Verda, MD, PhD – j

Abe Wandersman, PhD -- k

**Co-Investigators**

Michael Berbaum, PhD -- f

Kishore Bobba, MD – j

Joe Colla, MD -- g

Kim Erwin, MDes -- d

Andrea Lamont, PhD -- k

Molly Martin, MD. MAPP -- b

Sarah Norell, MDes, MFA -- d

Ananta Pandit, MD – j

Kay Saving, MD -- h

Robin Shannon, DNP, RN -- e

Robert Winn, MD -- c

Leslie Zun, MD -- j

**Research Staff**

Taif Hassan, MD -- a

Patricia Lasley, MPH – j

Kristin Monnard, MPH – i, j

Judith Nocek, PhD -- a

Pamela Roesch, MPH – i, j**Affiliation**

1. University of Illinois at Chicago, Division of Hematology and Oncology
2. University of Illinois at Chicago, Department of Pediatrics
3. University of Illinois at Chicago, Division of Pulmonary, Critical Care, Sleep and Allergy, Associate Vice Chancellor for Population Health Sciences
4. Program for Healthcare Delivery Design, Population Health Sciences Program, Office of the Vice Chancellor for Health Affairs
5. University of Illinois at Chicago, College of Nursing
6. University of Illinois at Chicago, Department of Epidemiology and Biostatistics
7. University of Illinois Health, Department of Emergency Medicine
8. University of Illinois College of Medicine, Peoria
9. Sinai Urban Health Institute
10. Sinai Health System
11. University of South Carolina
